# Duration-dependent physiological, perceptual, and technical changes during a 2-hour low-intensity training session in female cross-country skiers

**DOI:** 10.3389/fphys.2025.1534858

**Published:** 2025-02-20

**Authors:** Per-Øyvind Torvik, Guro Strøm Solli, Rune Kjøsen Talsnes, Øyvind Sandbakk

**Affiliations:** ^1^ Department of Sports Sciences and Physical Education, Nord University, Levanger, Norway; ^2^ Department of Neuromedicine and Movement Science, Faculty of Medicine and Health Sciences, Centre for Elite Sports Research, Norwegian University of Science and Technology, Trondheim, Norway; ^3^ School of Sport Science, UiT The Artic University of Norway, Tromsø, Norway

**Keywords:** long-distance slow training, duration, intensity, G2 and G3 technique, XC skiing

## Abstract

**Purpose:**

This study investigated duration-dependent physiological, perceptual, and technical changes during a 2-hour low-intensity training (LIT) session in female cross-country (XC) skiers.

**Methods:**

Twelve national-level female XC skiers (age:21.4 ± 2.7 years, maximal oxygen uptake: 58.1 ± 5.3 mL min⁻^1^·kg⁻^1^) performed a 2-hour LIT session, roller-ski skating in the laboratory while alternating between two main sub-techniques (Gear-2 and Gear-3). Acute physiological and perceptual responses, including oxygen uptake, carbon dioxide production, ventilation, breathing frequency, respiratory exchange ratio, blood lactate concentration, and rate of perceived exertion, as well as kinematic variables (cycle length and cycle rate), were collected at four consecutive timepoints (T1–T4) for both sub-techniques. A post-session questionnaire collected data on perceived total, ventilatory, and mental exertion as well as perceived training quality.

**Results:**

Relatively small duration-dependent changes were observed for most of the physiological measures. The most pronounced changes were a main effect of time on breathing frequency (BF; F (3,30) = 3.52, ηp2 = 0.260, *P* = 0.027) and rate of perceived exertion (RPE; F (3, 33) = 4.43, ηp2 = 0.287, *P* = 0.010). More specifically, BF was higher in Gear-3 at both T2 (45.5 ± 7.7 breaths·min^-1^) and T3 (45.5 ± 7.7), compared to T1 (43.2 ± 7.3, all *P* < 0.05). Furthermore, the rate of perceived exertion was higher in both Gear-2 and Gear-3 at T2 (G2: 12.1 ± 1.0, G3: 11.2 ± 1.6) and T3 (G2: 12.2 ± 1.1, G3: 11.2 ± 1.6), compared to T1 (G2: 11.5 ± 1.2, G3: 10.6 ± 1.2, all *P* < 0.05). No differences were observed in kinematic variables (cycle length and cycle rate) between T1 - T4. Lastly, no differences in perceived total, ventilatory, and mental exertion, as well as perceived training quality, were observed between the first and second half of the LIT session.

**Conclusion:**

Well-trained female XC skiers performed a 2-hour LIT session while roller-ski skating in the laboratory with relatively small duration-dependent physiological, perceptual, and technical changes.

## Introduction

Cross-country (XC) skiing is a demanding winter endurance sport. The varied terrain and constantly changing speeds require alternating between four to five different sub-techniques in each of the main styles (classic and skating) and different loading of the upper and lower body (Sandbakk and Holmberg, 2014; Sandbakk and Holmberg, 2017; Losnegard, 2019; Pellegrini et al., 2021). Specifically for the skating style, these sub-techniques include Gear-1 (used for steep uphill terrain), Gear-2 (moderate inclines), Gear-3 (flat terrain and slight inclines), Gear-4 and 5 (higher speeds) (Pellegrini et al., 2021). This study focuses on Gear-2 and Gear-3, which are most commonly used during prolonged low-intensity training sessions. To meet the competitive demands, XC skiers perform high annual training volumes (∼750–950 h), with approximately 90% of the training dedicated to endurance training and 10% to speed and strength training (Sandbakk et al., 2011; Tønnessen et al., 2014; Sandbakk et al., 2016; Solli et al., 2017). Most of the endurance training (approximately 90%) is performed as LIT, which aligns with the patterns observed in other endurance sports (Seiler, 2010) and constitutes a pivotal training stimulus for physiological and technical development (Laursen, 2010; Sandbakk and Holmberg, 2017; Talsnes et al., 2022).

The high volumes of LIT performed by XC skiers are distributed across sessions of varying duration, performed in both specific modes (i.e., skiing and roller-skiing in the skating and classical style) and general modes (i.e., running and cycling). For example, a case study of the world’s most decorated XC skier revealed that she allocated 17% of her training to LIT sessions of <90 min, 24% to sessions of >150 min, while the majority (59%) fell within the range of 90–150 min (Solli et al., 2017). Despite the available literature underpinning substantial volumes of LIT as a part of the development process in XC skiers and endurance athletes in general, acute and chronic responses to LIT remain largely unexplored (Sandbakk, 2023).

The structure of LIT sessions (i.e., manipulation of volume and frequency) may influence acute and chronic responses. For instance, duration-dependent drifts in heart rate (HR) and the rate of perceived exertion (RPE) are commonly associated with prolonged sessions and modulated by exercise duration (Maunder et al., 2021). Furthermore, XC skiing performance is not only influenced by the skier’s endurance capacity but also by skiing technique (Sandbakk and Holmberg, 2017). However, the ability to maintain or alter the temporal patterns and kinematic variables of different sub-techniques during prolonged LIT sessions remains unexplored despite its potential significance for technical quality and overall performance development.

In contrast to the available literature on acute and chronic responses from high-intensity training (HIT) in endurance sports, LIT and particularly long-duration LIT sessions have been sparsely examined (Hofmann and Tschakert, 2017; Sandbakk, 2023). In this context, both the timing and magnitude of physiological, perceptual, or technical changes during LIT sessions may offer valuable insights. Given the substantial quantity and proportion of LIT training in XC skiing, even minor miscalculations of this training stimulus could significantly influence the effectiveness of LIT training in eliciting maladaptation versus positive adaptation, ultimately affecting performance development.

Therefore, to increase our understanding of LIT in XC skiing, this study aimed to investigate duration-dependent physiological, perceptual, and technical changes during a 2-hour LIT session in national-level female XC skiers. We hypothesized that during the 2-hour LIT session, there would be a duration-dependent significant increase in perceived exertion, physiological responses, while kinematic variables would remain stable.

## Methods

### Participants

Twelve national-level Norwegian female XC skiers participated in the study (mean ± standard deviation [SD]: age: 21.4 ± 2.7 years, body height: 165.2 ± 5.1 cm, body mass: 61.8 ± 8.2 kg, maximal heart rate (HR_max_): 199.6 ± 6.5 beats·min^-1^, FIS points: 192.2 ± 64.3, maximal oxygen uptake (VO_2max)_: 58.1 ± 5.3 mL·min^-1^·kg^-1^, annual training volume: 611 ± 123 h). The performance and VO_2max_ values of the athletes included in this study are at the level of Tier three national level female XC skiers (McKay et al., 2022). All participants performed more than five endurance training sessions per week (8–20 hours/week), with a mean annual training duration of 611 ± 123 h. Before providing written consent to participate, all participants were informed about the study’s content. The Regional Committee for Medical and Health Research Ethics waives requirements for ethical approval for such studies and the ethics was therefore performed according to the institutional requirements at Nord University. Approval for data security and handling was obtained from the Norwegian Centre for Research Data (reference 421,018). The study adhered to the principles outlined in the Declaration of Helsinki.

### Design

All participants performed two initial incremental tests to exhaustion using the Gear-2 (G2) and Gear-3 (G3) sub-techniques of the skating style. These tests were performed in a counterbalanced order on the same day to determine maximal oxygen uptake (VO_2max_) and maximal speed (V_max_). Furthermore, a 2-hour LIT session on a separate day within 2 weeks from the initial test was performed by all participants. The LIT session was performed at a roller-ski treadmill in the laboratory, where the athletes alternated between the two main sub-techniques (G2 and G3) of the skating style. Physiological, perceptual, and technical responses were collected during the session.

### Incremental tests

Participants were instructed to adhere to the regular training routines and nutritional protocols they would follow before a competition. Following a standardised 10-minute warm-up, an incremental test to exhaustion was performed to determine VO_2max_ and V_max_. The test was performed at a constant incline (8% for G2 and 3% for G3). The G2 test started at 6 km·h^-1^ with a 1 km·h^-1^ increase every minute, while the G3 test started at 12 km·h^-1^ with a 2 km·h^-1^ increase every minute until voluntary exhaustion. The tests were separated by 20 min of active rest at low intensity. The test ended when the skiers could no longer maintain their speed while crossing a mark in the middle of the treadmill with their roller-ski wheels. V_max_ was calculated as V_max_ = Vc + ((tfinal/60)*ΔV), with Vc representing the speed of the last completed workload, tfinal indicating the duration of the last workload, and ΔV denoting the change in speed between each workload. VO_2max_ was defined as the average of the two highest and consecutive VO_2_ measurements averaged over a 30-second period, while maximal heart rate (HR_max_) was recorded as the highest HR observed during a 5-second interval. All participants were familiarised with treadmill roller-ski skating and the test protocol before testing.

### LIT session

**FIGURE 1:**
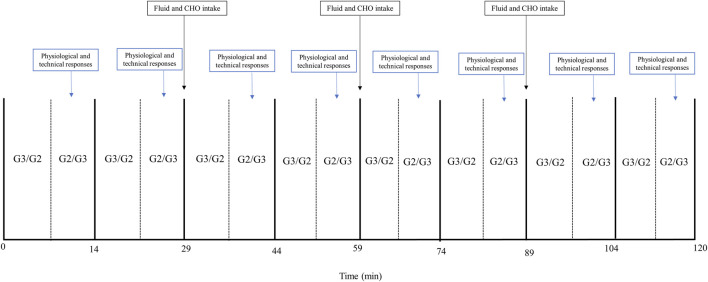
Schematic overview of the LIT session used in the study. G3: Gear-3 skating sub-technique at 3% incline; G2: Gear-2 skating sub-technique at 8% incline; VO_2_: Oxygen uptake; Bla: Blood lactate concentration; RPE: Rate of perceived exertion; CHO: carbohydrate.

The initial incremental tests were employed to determine the intensity of the 2-hour LIT session. The LIT session alternated between the G2 and G3 sub-techniques, both performed at an intensity corresponding to 65% of V_max_ from the incremental tests. This workload corresponds with the definition of LIT, characterized by workloads below ventilatory threshold 1 (VT1), <2 mM blood lactate concentration (Bla), 60%–82% of HR_max_, 9–12 on the RPE scale (Foster, 1998), and VO_2_ ranging from 65% to 75% of VO_2max._ The 2-hour LIT session comprised 16 x 7-minute stages, with counterbalanced alternation between the G2 and G3 sub-techniques. A pilot study, which included two female skiers, was conducted before the data collection to verify the protocol’s feasibility and alignment with the participants’ daily training. Incline settings of 3% in G3 and 8% in G2 were chosen to ensure appropriate technique and muscular loading, with the selection of G2 and G3 techniques reflecting the preferred modalities at these speed-incline combinations (Losnegard, 2019). Acute physiological and perceptual responses, including VO_2_, carbon dioxide production (VCO_2_), ventilation (VE), breathing frequency (BF), respiratory exchange ratio (RER), Bla, RPE and kinematic variables (cycle length [CL] and cycle rate [CR]) were measured at four consecutive timepoints (T1–T4). T1, T2, T3, and T4 correspond to 14, 29, 44 and 59 min for the first half, and 74 and 89, 104 and 120 min for the second half of the LIT session, respectively (Figure 1)*.* The participants consumed 2 dL of sports drink (Maxim Fresh lemon flavour, containing 70 g of carbohydrates per litre) every 29 min. The test protocol is presented in Figure 1.

To evaluate their experiences performing the LIT session, the participants completed a post-session questionnaire. This questionnaire collected data on perceived total, ventilatory, and mental exertion as well as their perceived training quality on a scale ranging from one (poor) to 10 (excellent) from a physical, technical, and mental perspective using the training quality scale recently developed by (Shell et al., 2023).

### Instruments and materials

All tests were conducted on a motor-driven treadmill (Rodby 3500 ML, Södertalje, Sweden) featuring a non-slip rubber belt. Participants used classic roller-skis equipped with standard category-2 resistance wheels (IDT Sports, Lena, Norway). Participants used their own poles fitted with special carbide tips to ensure optimal grip on the treadmill. During the incremental tests, participants were secured with a safety harness connected to the treadmill’s emergency brake. Respiratory variables were measured using open-circuit indirect calorimetry equipped with a mixing chamber (Vyntus CPX, CareFusion, Höchberg, Germany). Before each test session, the instruments were calibrated against ambient air conditions and certified gases of known concentrations (O_2_ 15.0%; CO_2_ 5.0%). The flow transducer (Vyntus CPX, Vyaire Medical, Mettawa, IL, United States) was calibrated using a 3-L high-precision calibration syringe (Calibration Syringe D, SensorMedics, Yorba Linda, CA, United States). HR was continuously monitored using a Garmin Forerunner 920 XT. Bla concentration and glucose levels were measured using the stationary Biosen C-Line lactate analyser (Biosen, EKF Industrial Electronics, Magdeburg, Germany) with 20 μL of blood drawn from the fingertip. Athletes’ body masses and heights were measured using a medical scale and stadiometer (Seca model 708, Seca GmbH, Hamburg, Germany).

Temporal patterns were assessed using 2-D video recorded with an Apple iPad 4 (MD791 KN/A United States) at 30 frames per second. CL and CR were analysed using Onform software (version 2.03 United States). The iPad was positioned at a 90° angle to the skiing direction on the skiers’ left side, 4.25 m from the centre of the skiing treadmill. Average cycle characteristics were determined by timing 10 stable cycles in three different sections of each video, during both the initial test and the LIT session. Cycle time (CT) was measured from the time between two pole plants on the left side. CL was calculated by multiplying the treadmill’s speed by the CT, while CL was obtained as the reciprocal of CT.

### Statistical analysis

The Statistical Package for the Social Sciences (SPSS 26.0, IBM Corp., Armonk, NY, United States) was used for statistical analyses, with significance set at P < 0.05. Data are presented as mean ± standard deviation (SD). Normality was assessed by visual inspection of histograms, and Mauchly’s test was used to examine the assumption of sphericity. In case of violated sphericity (epsilon <0.75), the degrees of freedom were corrected using the Greenhouse–Geisser correction. Differences between G2 and G3 in the incremental tests were assessed using a paired-samples t-test. A two-way repeated measures ANOVA was employed to examine the effects of sub-technique and time. Subsequent one-way repeated ANOVA tests identified significant differences between timepoints. Independent-samples t-tests were conducted for comparisons involving only two means (e.g., sub-techniques at a given timepoint). Non-normally distributed variables were assessed using the Wilcoxon signed-rank test. Effect sizes were calculated using partial Eta square (ηp2), with values > 0.01, 0.06, and 0.14 indicating small, moderate, and large effects, respectively (Cohen, 2013).

## Results

No differences between the G2 and G3 sub-techniques in peak physiological measures during the initial incremental tests were observed, except for 6.4% ± 7.1% higher VE in G3 than in G2 (Table 1).

**TABLE 1:** Physiological, perceptual, and technical responses during incremental tests to exhaustion in the two main sub-techniques of the skating style (G2 and G3) in national-level female cross-country skiers (n = 12).

	G2	G3	P-value
HR_peak_ (beat·min^-1^)	191.1 ± 6.3	191.1 ± 6.0	1.000
VO_2peak_ (L·min^-1^)	3.61 ± 0.36	3.60 ± 0.41	0.990
VO_2peak_ (mL·kg^-1^·min^-1^)	58.1 ± 5.3	58.1 ± 5.5	0.968
VE (L·min^-1^)	116.9 ± 12.4	124.2 ± 13.2	0.011
RER	1.041 ± 0.052	1.044 ± 0.040	0.754
Blood lactate (mmol·L^-1^)	8.96 ± 2.09	9.38 ± 2.06	0.328
RPE (6–20)	17.8 ± 1.5	18.3 ± 1.3	0.137
V_max_ (km·h^-1^)	13.5 ± 1.0	21.4 ± 1.1	0.000
Cycle length (m)	4.3 ± 0.3	5.3 ± 0.3	0.000
Cycle rate (Hz)	67.8 ± 4.3	68.0 ± 3.6	0.920

**FIGURE 2:**
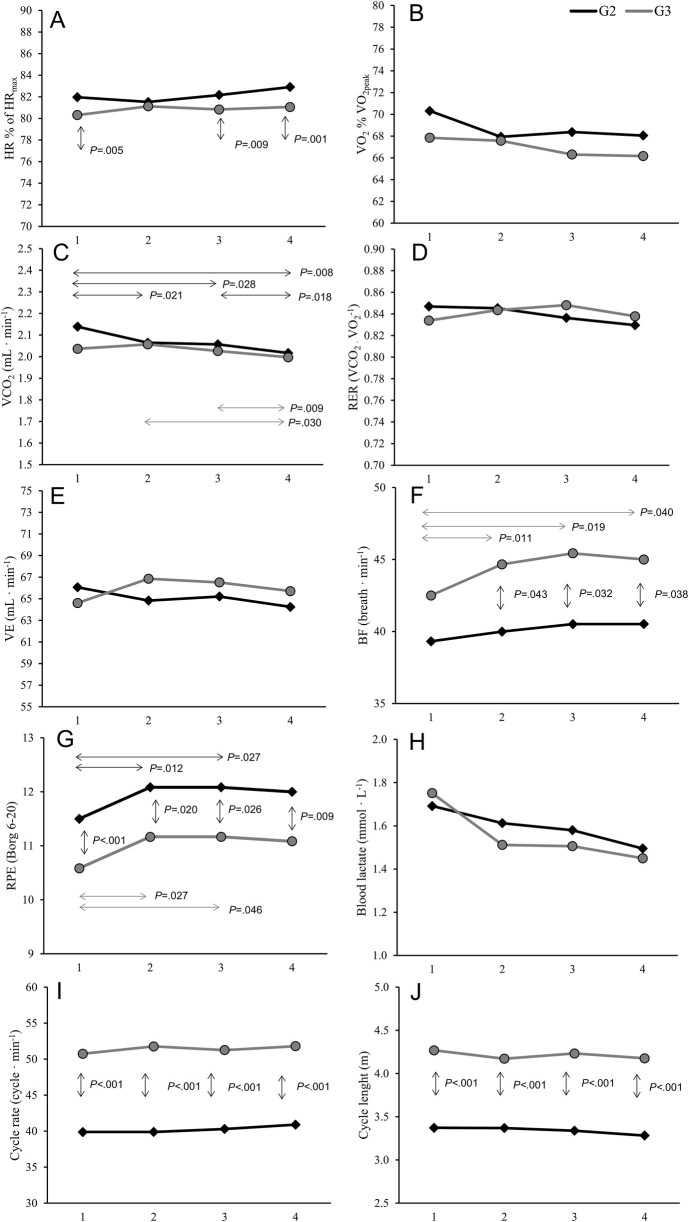
Physiological, perceptual, and technical responses to a 2-hour low-intensity training session in national-level female cross-country skiers: heart rate **(A)**; VO_2,_ oxygen uptake **(B)**; CO_2,_ carbon dioxide uptake **(C)**; RER, respiratory exchange ratio **(D)**; VE, ventilation **(E)**; BF, breathing frequency **(F)**; RPE, rate of perceived exertion **(G)**; blood lactate concentration **(H)**; cycle rate **(I)**; cycle length **(J)**. ↕Significantly different between sub-techniques (G2 and G3). ↔Significantly different between timepoints (T1–T4).

Physiological, perceptual, and technical responses to the 2-hour LIT session are presented in Table 2 and Figure 2. A significant interaction effect between sub-technique and time was observed for VCO_2,_ F (3, 30) = 7.69, ηp2 = 0.435, *P* < 0.001; RER, F (3, 30) = 7.13, ηp2 = 0.416, *P* < 0.001; and VE, F (3, 30) = 6.67, ηp2 = 0.400, *P* = 0.001. For VCO_2_, there was a significant effect of time in both G2, F (3, 30) = 7.70, *P* = 0.009, and G3, F (3, 33) = 3.79, *P* = 0.019, with VCO_2_ being 0.8%, 1.1%, and 3% lower in G2 at T1 compared to T2, T3, and T4, respectively, and 1.9% lower at T4 compared to T3. In G3, VCO_2_ was 1.5% and 1.4% lower at T4 compared to T2 and T3, respectively (all *P* < 0.05; Figure 2). The RER values were 1.3% lower in T3 compared to T4 (*P* = 0.044) in G3, and for VE, 3.5% and 3% higher values were observed in G3 at T2 and T3 compared to T1, respectively (all *P* < 0.05; Figure 2). No significant differences between G2 and G3 were detected at any of the four timepoints for VCO_2_, RER, or VE.

**TABLE 2:** Physiological, perceptual, and technical responses to 2-hour low-intensity training session in national-level female cross-country skiers (n = 12).

Sub-technique	G2				G3				ANOVA *p*-Value (ηp2)		
Timepoints	1	2	3	4	1	2	3	4	Sub-technique	Time	Sub-tech x time
HR (beat·min-1)	163.4 ± 9.0	162.6 ± 7.5	163.8 ± 8.5	165.3 ± 7.0	160.2 ± 10.0	161.8 ± 8.1	161.2 ± 7.8	161.7 ± 7.5	0.005 (0.524)	0.380 (0.088)	0.052 (0.207)
%HR_max_	82.0 ± 5.4	81.5 ± 4.2	82.2 ± 5.0	82.9 ± 4.2	80.3 ± 5.3	81.1 ± 4.9	80.8 ± 4.6	81.1 ± 4.1	0.005 (0.530)	0.397 (0.085)	0.052 (0.206)
VO_2_ (mL· kg^-1^· min^-1^)	40.9 ± 4.3	39.6 ± 3.5	39.9 ± 3.6	39.7 ± 3.4	39.6 ± 4.1	39.2 ± 3.1	38.6 ± 3.1	38.4 ± 3.0	0.143 (0.201)	0.177 (0.169)	0.165 (0.154)
VO_2_ (L· min^-1^)	2.51 ± 0.38	2.43 ± 0.28	2.44 ± 0.29	2.43 ± 0.26	2.44 ± 0.41	2.41 ± 0.35	2.37 ± 0.32	2.36 ± 0.33	0.207 (0.154)	0.177 (0.168)	0.158 (0.157)
%VO_2max_	70.3 ± 9.1	68.1 ± 7.2	68.4 ± 6.1	68.1 ± 5.9	68.0 ± 6.7	67.5 ± 5.9	66.4 ± 5.1	66.1 ± 5.8	0.077 (0.279)	0.179 (0.168)	0.150 (0.160)
VCO_2_ (L· kg^-1^· min^-1^)	2.13 ± 0.31	2.06 ± 0.25	2.05 ± 0.23	2.02 ± 0.22	2.03 ± 0.29	2.04 ± 0.29	2.02 ± 0.25	1.98 ± 0.25	0.342 (0.090)	0.009 (0.435)	0.006 (0.333)
RER	0.850 ± 0.05	0.846 ± 0.03	0.838 ± 0.03	0.830 ± 0.03	0.837 ± 0.05	0.845 ± 0.03	0.850 ± 0.02	0.839 ± 0.02	0.736 (0.012)	0.641 (0.054)	0.007 (0.416)
BF	39.6 ± 2.3	40.0 ± 2.4	40.5 ± 2.6	40.5 ± 3.8	43.2 ± 7.3	45.5 ± 7.7	46.1 ± 7.3	45.5 ± 8.2	0.034 (0.377)	0.027 (0.260)	0.179 (0.149)
VE (L· min^-1^)	66.1 ± 8.7	64.8 ± 7.6	65.0 ± 8.0	64.2 ± 8.3	64.8 ± 11.5	66.8 ± 11.3	66.5 ± 10.0	65.5 ± 11.3	0.574 (0.033)	0.484 (0.077)	0.001 (0.400)
Blood lactate (mmol·L^-1^)	1.69 ± 0.50	1.61 ± 0.54	1.58 ± 0.59	1.49 ± 0.65	1.75 ± 0.75	1.51 ± 0.43	1.51 ± 0.61	1.45 ± 0.55	0.421 (0.060)	0.107 (0.166)	0.681 (0.044)
RPE (6–20)	11.5 ± 1.2	12.1 ± 1.0	12.1 ± 1.0	12.0 ± 0.9	10.6 ± 1.2	11.2 ± 1.6	11.2 ± 1.6	11.1 ± 1.4	0.003 (0.556)	0.010 (0.287)	0.975 (0.011)
CL (m)	3.37 ± 0.23	3.37 ± 0.24	3.34 ± 0.25	3.28 ± 0.24	4.27 ± 0.22	4.17 ± 0.30	4.23 ± 0.43	4.18 ± 0.25	<0.001 (0.942)	0.291 (0.106)	0.510 (0.067)
CR (Hz)	39.9 ± 2.9	39.8 ± 2.9	40.3 ± 2.1	40.9 ± 2.7	50.8 ± 3.1	51.8 ± 3.4	51.3 ± 4.0	51.8 ± 2.5	<0.001 (0.959)	0.364 (0.091)	0.654 (0.047)

The values are means ± SD; HR, heart rate; %HR_max,_ percentage of maximal heart rate; VO_2_, oxygen uptake; %VO_2peak_, percentage of peak oxygen uptake; VCO_2_, volume carbon dioxide consumption; RER, respiratory exchange ratio; BF, breathing frequency; VE, ventilation; RPE, rate of perceived exertion; CL, cycle length; CR, cycle rate; ANOVA, analysis of variance; ηp2, partial Eta square effect size; NS, not significant; 1,2,3,4 timepoints.

There was a significant main effect of time on BF, F (3, 30) = 3.52, ηp2 = 0.260, *P* = 0.027, and RPE, F (3, 33) = 4.43, ηp2 = 0.287, *P* = 0.010. Analyses revealed 5.1% and 6.9% higher BF in G3 at T2 and T3, respectively, compared to T1. Furthermore, 5.1% and 5.5% higher RPE in both G2 and G3 were found at T2 and T3, respectively, compared to T1 (all *P* < 0.05; Figure 2). Although not statistically significant, effect sizes indicated a large effect of time on VO_2_ and Bla response and a moderate effect of time on HR and VE over the four timepoints. No significant main effect of time on either CL or CR was observed, although effect sizes indicated a moderate effect of time on CR and CL.

A significant main effect of sub-technique was observed on HR, BF, CL, and CR (Table 2). Analyses revealed 1.7%–2.3% lower HR when using G3 compared to G2 at timepoints 1, 3, and 4 (P < 0.009; Figure 2). RPE values were 8% lower when using G3 than G2 at all timepoints (P < 0.020; Figure 2). Conversely, 10%–11% higher BF was observed when using G3 compared to G2 at timepoints 2, 3, and 4 (P < 0.043; Figure 2). Regarding temporal patterns, 19%–21% longer CL and 21%–23% higher CR were observed in G3 compared to G2 at all timepoints (P < 0.001; Figure 2).

**FIGURE 3:**
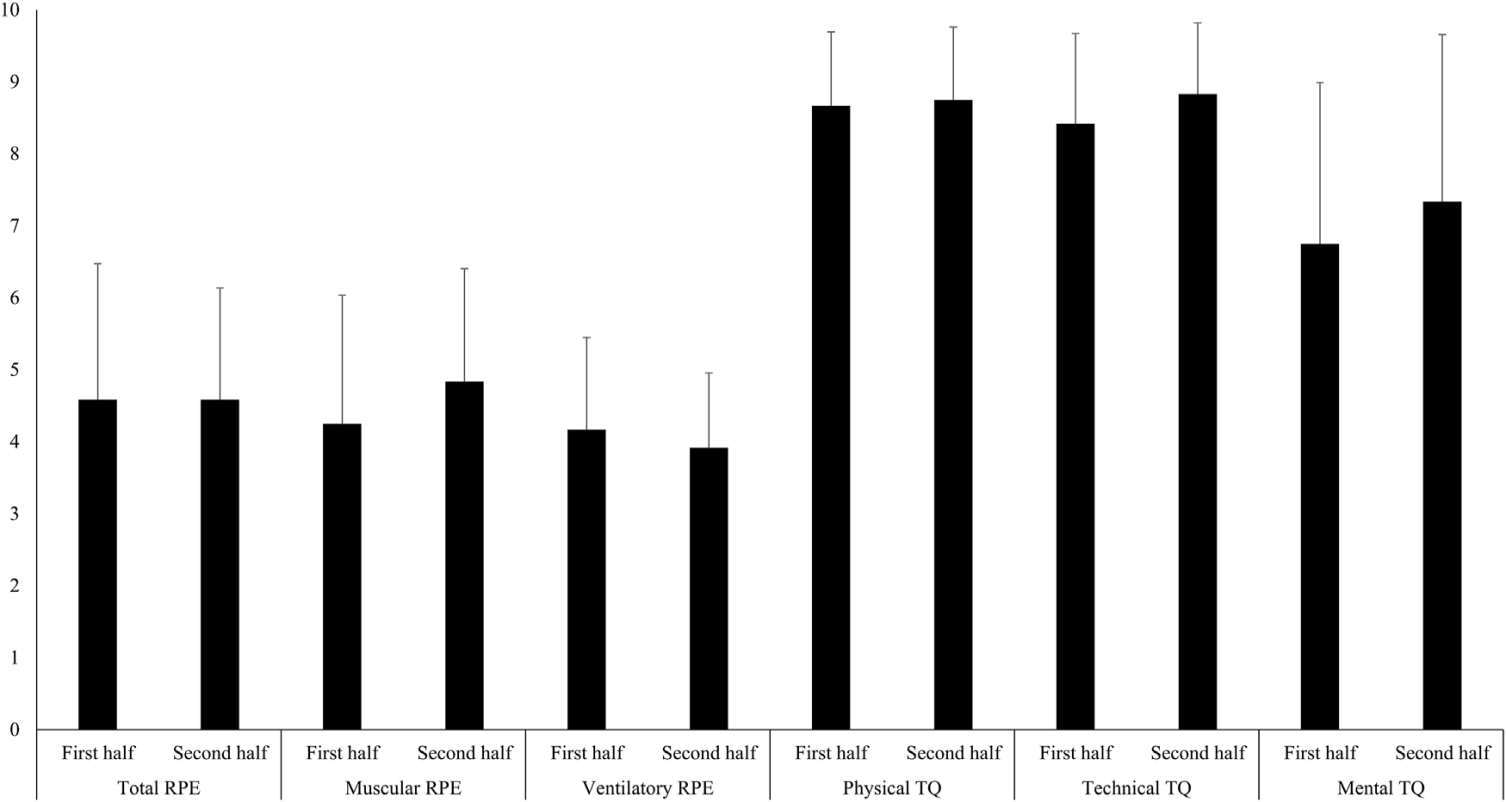
Rate of perceived total, muscular, and ventilatory exertion, as well as perceived physical, technical, and mental training quality for the first and second half of the 2-hour low-intensity session in national-level female cross-country skiers (n = 12).

An overview of the participants’ perceived total, muscular, and ventilatory RPE as well as their rate of physical, technical, and mental training quality is presented in Figure 3. No significant differences in either of the RPE measures or perceived training quality were observed between the first and second half of the LIT session.

## Discussion

This study investigated duration-dependent physiological, perceptual, and technical changes during a 2-hour LIT session in female XC skiers. The main findings are as follows: 1) most physiological measures did not change substantially during the LIT session, although breathing frequency and RPE were slightly increased; and 2) no differences were observed in kinematical variables nor perceived exertion and training quality between the first and second half of the LIT session.

The present study was conducted to model potential duration-dependent perceptual, physiological, and technical changes during a 2-hour LIT training session for XC skiers. Most endurance training (approximately 90%) in XC skiing comprises LIT, and training sessions similar to those employed in the study are used multiple times per training week throughout an annual training cycle (Stöggl and Sperlich, 2014; Sandbakk and Holmberg, 2017; Solli et al., 2017). Although some intensity measures increased slightly throughout the session, most measures were unchanged. Notably, both VO2 and HR remained relatively stable, in contrast to previous studies reporting cardiovascular drift in HR during prolonged endurance sessions (Maunder et al., 2021; Smyth et al., 2022). The reasons for the lack of physiological drift in intensity measures in our study are unknown but could be related to the design of the experimental protocol or the participants’ nutritional intake, which prevented dehydration and glycogen depletion during the session. Overall, we observed only minor changes in the physiological, perceptual, and technical changes, suggesting that well-trained female XC skiers can maintain internal intensity during a 2-h LIT session. Previous studies have shown that high volumes of LIT, comprising 80%–90% of the total training volume, are a common feature across most Olympic endurance sports. This underscores the importance of LIT for success in endurance sports (Tønnessen et al., 2024). Supporting this, Hofmann and Tschakert (2017) observed that LIT in triathlon facilitated endurance adaptations with minimal fatigue accumulation. The findings of the current study align with those of Maunder et al. (2021), who demonstrated relatively stable physiological parameters during prolonged LIT sessions in cycling. Collectively, these results reinforce the notion that high volumes of LIT are essential for endurance athletes, enabling them to accumulate substantial training volumes while maintaining low fatigue levels, which in turn supports high-quality performance in key intensive sessions.

Despite relatively similar physiological responses throughout the session, some changes in internal intensity measures were evident. This was particularly evident for BF, with a 5%–7% increase during the session, although no changes in VE were observed, suggesting different developments in BF and total volume. This finding aligns with recent studies suggesting that BF and tidal volume are regulated by different mechanisms during endurance exercise (Nicolò et al., 2017a; Nicolò et al., 2017b), with BF regarded as a more crucial indicator of physical effort and fatigue. Moreover, RPE values in the current study tended to follow the same pattern as BF with a slight increase as the session progressed. Markedly, the increase in BF was only significant in the G3 sub-technique. The reason for this finding is unknown, but previous research has observed distinct ventilatory responses between running and cycling, which may be attributed to the coupling intensity between locomotion and BF in these two exercise modalities (Elliott and Grace (2010). Based on this, it could be speculated that the different temporal patterns in the G3 versus G2 sub-techniques may affect BF. However, more research is needed to understand duration-dependent changes in BF during prolonged endurance exercise both in XC skiing and endurance sports in general.

A vital part of the training stimuli of LIT sessions involves performing a high number of sport-specific repetitions. Therefore, detecting potential duration-dependent changes in technique during LIT sessions is relevant. Although no significant differences in kinematical variables were observed in the G2 or G3 sub-technique, effect sizes indicated small changes in both CR and CL, with a pattern of increased CR and reduced CL throughout the session. This change could be relevant because of its effect on technical execution during the high number of cycle repetitions during XC skiing races. Still, the skiers were able to maintain their technical patterns relatively well throughout the 2-hour LIT session, which was also confirmed in the questionnaires; the athletes perceived no differences in technical quality when comparing the first versus the second part of the LIT session.

Although most internal intensity measures indicated that the LIT session was performed at an intensity within the LIT domain, the female participants in this study exhibited a higher average HR than expected. Despite that factors such as hydration, temperature, air conditions, and familiarity with treadmill skiing, did not seem to account for the observed phenomenon. Previous studies by McGawley et al. (2017) and Solli et al. (2018) have reported that female XC skiers tend to display higher relative HR during classic LIT training than men. However, further research is needed to better understand the relationship between HR and other internal intensity measures among female XC skiers.

The strength of this study is the inclusion of well-trained, national-level female XC skiers, providing valuable insights into an understudied population in endurance training research. Additionally, the controlled laboratory setting enabled precise monitoring of physiological, perceptual, and technical variables during prolonged LIT sessions. However, the study also has some limitations. This includes the relatively small sample size limiting the generalizability. Furthermore, the roller-skiing setup in the laboratory environment may differ from outdoor skiing conditions, potentially reducing the ecological validity of the results.

### Practical applications

The relatively small changes observed in physiological, perceptual, and technical markers throughout the LIT session suggest that well-trained XC skiers can perform multiple 2-hour LIT sessions without accumulating significant fatigue. As a result, engaging in high volumes of this type of training appears feasible without compromising the quality of essential sessions, such as high-intensity or prolonged low-intensity endurance training.

## Conclusion

Well-trained female cross-country skiers performed a 2-hour LIT session while roller-ski skating in the laboratory with relatively small duration-dependent physiological, perceptual, and technical changes. These results underscore the possibility to endure high volumes of this type of training in the pursuit of achieving endurance performance improvements.

## Data Availability

The raw data supporting the conclusions of this article will be made available by the authors, without undue reservation.
